# Applicability of Dynamic Energy Budget (DEB) models across steep environmental gradients

**DOI:** 10.1038/s41598-018-34786-w

**Published:** 2018-11-06

**Authors:** Cristián J. Monaco, Christopher D. McQuaid

**Affiliations:** 1 Department of Zoology and Entomology, Rhodes University, Grahamstown, South Africa; 2 Present Address: Southern Seas Ecology Laboratories, School of Biological Sciences and The Environment Institute, The University of Adelaide, Adelaide, SA 5005 Australia

## Abstract

Robust ecological forecasting requires accurate predictions of physiological responses to environmental drivers. Energy budget models facilitate this by mechanistically linking biology to abiotic drivers, but are usually ground-truthed under relatively stable physical conditions, omitting temporal/spatial environmental variability. Dynamic Energy Budget (DEB) theory is a powerful framework capable of linking individual fitness to environmental drivers and we tested its ability to accommodate variability by examining model predictions across the rocky shore, a steep ecotone characterized by wide fluctuations in temperature and food availability. We parameterized DEB models for co-existing mid/high-shore (*Mytilus galloprovincialis*) and mid/low-shore (*Perna perna*) mussels on the south coast of South Africa. First, we assumed permanently submerged conditions, and then incorporated metabolic depression under low tide conditions, using detailed data of tidal cycles, body temperature and variability in food over 12 months at three sites. Models provided good estimates of shell length for both species across the shore, but predictions of gonadosomatic index were consistently lower than observed. Model disagreement could reflect the effects of details of biology and/or difficulties in capturing environmental variability, emphasising the need to incorporate both. Our approach provides guidelines for incorporating environmental variability and long-term change into mechanistic models to improve ecological predictions.

## Introduction

Improving our understanding and predictions of species responses to environmental variability requires mechanistic approaches capable of disentangling the relative and interacting effects of multiple drivers^1^. Because individuals can experience different conditions^2^, a focus at this level can help us to identify the relevant biological and ecological processes that drive the dynamics of populations and communities^3,4^. Classic energy budget models such as scope for growth have provided a means of quantifying the energy allocation and physiological state of organisms subject to specific biotic and abiotic conditions^5^. Although such models are informative of species’ adaptive capacities^6^, they are usually only applicable to the conditions defined by the experimental design. Given the inherent complexity and dynamism of natural systems, using the information gathered from these classic models to derive meaningful predictions for field conditions is difficult.

The introduction and ongoing development of the Dynamic Energy Budget (DEB) theory^7–9^, offers a mechanistic approach based on physical-chemical principles to integrating the physiological responses of organisms and quantifying *fitness-related traits* across time and space, and thus has gained much attention in the ecological literature. The scope and generality of DEB theory has been repeatedly confirmed empirically and theoretically, with a growing number of species being successfully parameterized^10^.

DEB models currently exist that can describe the effects of contrasting body temperature^11^, food availability^12^ or seawater pH^13^ on the physiological condition of animals throughout ontogeny. However, despite the capacity of DEB theory to incorporate contrasting physical conditions, the models are usually built and implemented for species/populations that experience relatively homogeneous environments, particularly in terms of food and temperature. For instance, marine mussels, which have long served as DEB theory model species^14^, are predominantly modelled under relatively stable subtidal conditions. While many DEB model applications have incorporated natural environmental variability, successfully capturing organisms’ physiological responses^15–17^, less attention has been given to explicitly testing model performance under steep gradients of environmental variability. Ignoring environmental variability can reduce the predictive power of energy budget models, and variability deserves special consideration when working with taxa subject to wide fluctuations in food and temperature, as recently highlighted for terrestrial and semi-aquatic species^18,19^.

**Figure 1:**
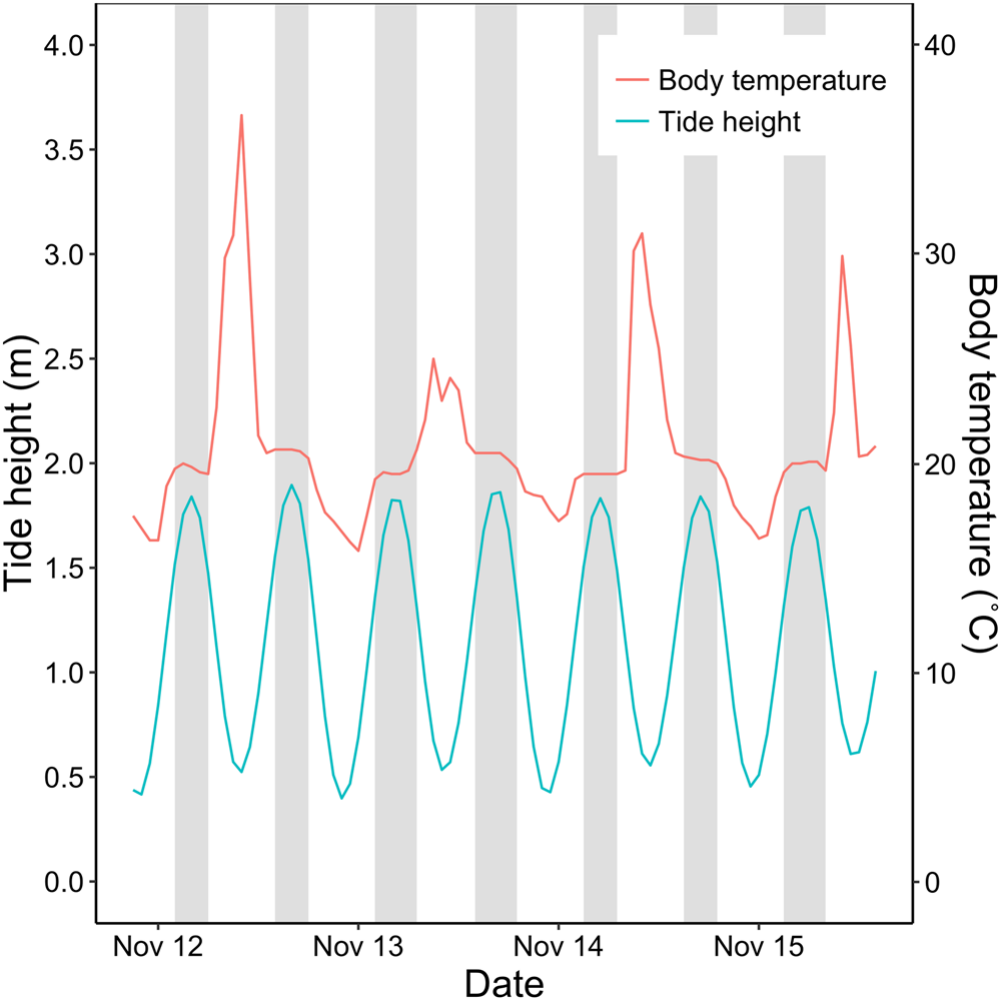
Example illustrating the changes in environmental variables and physiological state of high-shore intertidal organisms subjected to shifting tides. With the rise and fall of the tide (predictions by XTide), mussels experience alternating periods of submergence and aerial exposure (grey and white bars, respectively). This forces wide fluctuations in body temperature (recorded using robomussels), thermal sensitivity (e.g. metabolic depression), and intermittent windows of feeding and fasting.

Marine rocky shores form an exceptionally steep ecotone between permanently marine and permanently terrestrial conditions and are among the most variable of physical habitats^2,20,21^, making them ideal systems for testing the ability of DEB models to describe an organism’s physiological state under fluctuating conditions. With the ebb and flow of the tide, organisms experience regular changes in body temperature, desiccation, and food availability (Fig. 1). As almost all intertidal species are of marine origins, this results in a dramatic increase in physiological stress from low- to high-shore levels^22^.

This study examines the application of DEB models across the environmental stress gradient offered among shore levels using two model species: the mussels *Perna perna* and *Mytilus galloprovincialis* (hereafter *Perna* and *Mytilus*). *Perna* is indigenous to and *Mytilus* invasive on the south coast of South Africa, where they exhibit partial habitat segregation. Within the shore heights occupied by mussels, *Perna* dominates the lower and *Mytilus* the higher level, the two co-occurring in the middle^23^. This provides a good system to test the effectiveness of DEB models across a stress gradient that includes a high level of variability in physical conditions.

We use DEB models for South African populations of *Perna* and *Mytilus* to ask whether these models perform well for individuals found across a steep intertidal gradient of environmental forcing variables. To incorporate intertidal physical gradients into a DEB framework, we explicitly consider tidal dynamics and the associated fluctuations in body temperature and filter feeding^11^. Additionally, we incorporate dynamics in thermal sensitivity as, during aerial exposure, intertidal mussels can markedly depress their metabolic rates, often undergoing anaerobiosis^24–26^. These responses hinge on complex biochemical, physiological, and behavioural adaptations that differ widely among species^27–30^. However, with the aim of modelling energetics over a relatively long period (i.e. months to years), we make the simplifying assumption that during aerial exposure all processes associated with metabolic depression can be encapsulated by a constant empirically derived for each species.

## Materials and Methods

### DEB model topology

**Figure 2:**
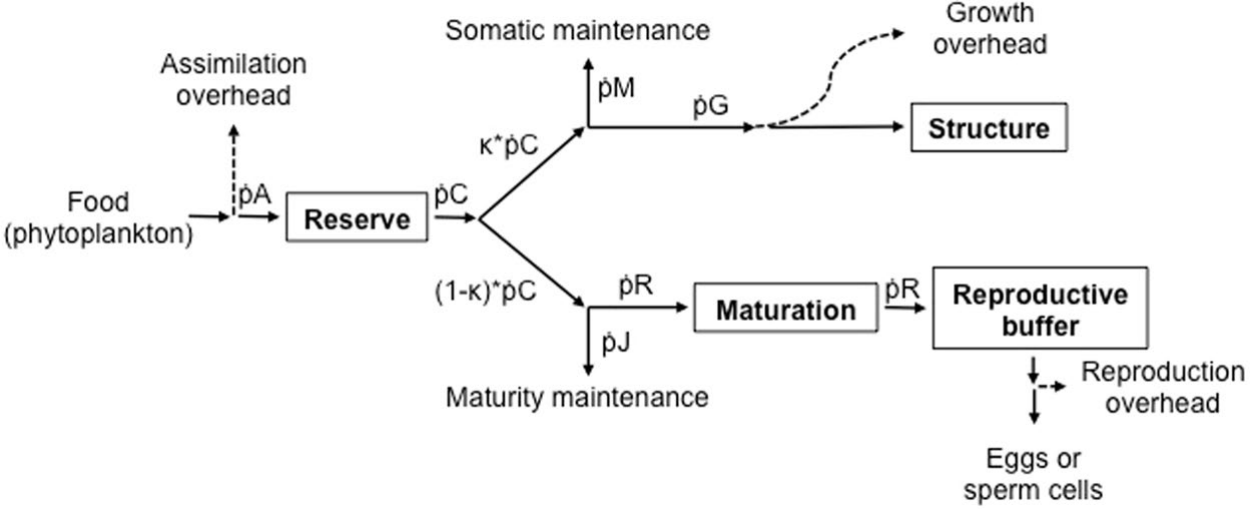
Schematic representation of the energy flows described by the Dynamic Energy Budget (DEB) model. State variables are illustrated in boxes. Arrows depict rates of energy flow: continuous lines = allocation to state variables and maintenance costs; dashed lines = loss of energy as overheads. Equations that describe the flows are given in Table 2.

We briefly describe the parameters and state variables that determine an organism’s physiological state. For details see Kooijman^8^ and Monaco, *et al*.^31^. We work with a modified standard DEB model: i.e. growth is isomorphic, except for the larval stage, when metabolic acceleration is implemented^32^, there is one reserve compartment and one structure compartment. The organism’s condition is defined by three state variables: amount of energy in reserve, E (J), volume of structure, V (cm^3^), and amount of energy in the reproductive buffer, ER (J) (Fig. 2).

Tables 1, 2 and 3 list the equations and parameters that describe flows of energy and the dynamics of state variables. Mussels acquire energy by ingesting phytoplankton, here assumed to be proportional to chlorophyll-a, X (μg L^−1^)^33^. Ingestion rate is described by ṗX (J d^−1^), which is constrained by the maximum surface-area specific ingestion rate parameter, {ṗ_Xm_} (J d^−1^ cm^−2^), and follows a scaled type II functional response, *f* (−), which in turn is controlled by the half-saturation coefficient, *X*_*k*_ (μg L^−1^). Assimilation rate, ṗ_A_ (J d^−1^), depends on the maximum surface-area specific assimilation rate, {ṗ_Am_} (J d^−1^ cm^−2^), the assimilation efficiency, *ae* (−), and the acceleration factor, *sM* (−), during the larval stage. The assimilated energy that is stored as reserves is utilized at a rate, ṗ_C_ (J d^−1^), and divided between somatic and reproductive allocation pathways in accordance to the kappa rule: a proportion *κ* (−) is used for somatic maintenance and growth (i.e. structure), while the rest, 1 − *κ*, goes towards maturity maintenance and reproduction (i.e. maturation or gametogenesis in adults). This implies that somatic processes have priority over allocation to reproduction. The energy used in somatic and maturity maintenance, ṗ_M_ and ṗ_J_ (J d^−1^) respectively, is proportional to structure. Growth occurs if *κ* * ṗ_C_ > ṗ_M_. Similarly, investment in reproduction, ṗ_R_ (J d^−1^), takes place if (1 − *κ*) * ṗ_C_ > ṗ_J_.

**Table 1:** Dynamic Energy Budget model equations that describe energy flows illustrated in Fig. 2.

Energy flow or state variable dynamics	Equation
Ingestion, ${\dot{{\rm{p}}}}_{X}$	${\dot{{\rm{p}}}}_{X}=\{{\dot{{\rm{p}}}}_{Xm}\}{V}^{2/3}f{T}_{C}$ $f=(\frac{X}{{X}_{K}+X})$
Assimilation, ${\dot{{\rm{p}}}}_{A}$	${\dot{{\rm{p}}}}_{A}=\{{\dot{{\rm{p}}}}_{Am}\}f{V}^{2/3}$ $\{{\dot{{\rm{p}}}}_{Am}\}=ae\{{\dot{{\rm{p}}}}_{Xm}\}{S}_{M}$ ${S}_{M}=\,{\rm{\min }}({V}^{1/3},{L}_{J})/{L}_{b}$
Utilization, ${\dot{{\rm{p}}}}_{C}$	${\dot{{\rm{p}}}}_{C}=\frac{E/V}{[{E}_{G}]+\kappa E/V}(\frac{[{E}_{G}]\{{\dot{{\rm{p}}}}_{Am}\}{V}^{2/3}}{[{E}_{m}]})+[{\dot{{\rm{p}}}}_{M}]V$
Somatic growth, ${\dot{{\rm{p}}}}_{G}$	${\dot{{\rm{p}}}}_{G}=\kappa \,{\dot{{\rm{p}}}}_{C}-{\dot{{\rm{p}}}}_{M}$
Somatic maintenance, ${\dot{{\rm{p}}}}_{M}$	${\dot{{\rm{p}}}}_{M}=[{\dot{{\rm{p}}}}_{M}]V{T}_{C}$
Reproduction/maturation, ${\dot{{\rm{p}}}}_{R}$	${\dot{{\rm{p}}}}_{R}=(1-\kappa ){\dot{{\rm{p}}}}_{C}-{\dot{{\rm{p}}}}_{J}$
Reproductive maintenance, ${\dot{{\rm{p}}}}_{J}$	${\dot{{\rm{p}}}}_{J}=\,{\rm{\min }}({\rm{V}},{V}_{P})[{\dot{{\rm{p}}}}_{M}](\frac{1-\kappa }{\kappa }){T}_{C}$
Reserve dynamics, E	$\frac{{\rm{dE}}}{{\rm{dt}}}={\dot{{\rm{p}}}}_{A}-{\dot{{\rm{p}}}}_{C}$
Structure dynamics, V	$\frac{{\rm{dV}}}{{\rm{dt}}}={\dot{{\rm{p}}}}_{G}/[{E}_{G}]$
Reproductive buffer dynamics, E_R_	$\frac{{{\rm{dE}}}_{R}}{{\rm{dt}}}={\dot{{\rm{p}}}}_{R}{\kappa }_{R}$
Temperature correction, *T*_*c*_	${T}_{C}=exp[\frac{{T}_{A}}{{T}_{1}}-\frac{{T}_{A}}{T}]{(1+exp[\frac{{T}_{AL}}{T}-\frac{{T}_{AL}}{{T}_{L}}]+exp[\frac{{T}_{AH}}{{T}_{H}}-\frac{{T}_{AH}}{T}])}^{-1}$ $${T}_{C}=\{\begin{array}{l}{T}_{C},\,when\,submerged\\ {T}_{C}{M}_{d},\,aerial\,exposure\end{array}\,$$

**Table 2:** Auxiliary equations to translate Dynamic Energy Budget model quantities (Table 1) to empirical biological metrics.

Biological metric	Equation
Shell length, L_W_	${{\rm{L}}}_{W}={V}^{1/3}/{\delta }_{M}$
Soma dry weight, W_sd_	W_*sd*_ = *Vd*_*V*_ + *Eρ*_*E*_
Gonad dry weight, W_gd_	W_*gd*_ = *E*_*R*_*ρ*_*E*_
Gonadosomatic index, GSI	${\rm{GSI}}={{\rm{W}}}_{gd}/{{\rm{W}}}_{sd}$

**Table 3:** Estimated Dynamic Energy Budget model parameters for *Perna perna* and *Mytilus galloprovincialis*.

Parameter	Symbol	Units	*Perna*	*Mytilus*
Surface-area specific ingestion rate	{ṗ_Xm_}	J d^−1^ cm^−2^	15.54	9.42
Half-saturation coefficient	*X*_κ_	μg L^−1^	0.50	2.10
Assimilation efficiency	ae	—	0.69	0.80
Fraction of energy used for growth and somatic maintenance	*κ*	—	0.82	0.47
Structural length at birth	*L*_*b*_	cm	0.0021	0.0019
Structural length at metamorphosis	*L*_*j*_	cm	0.0242	0.0197
Structural length at puberty	*L*_*p*_	cm	0.6729	0.6912
Volume-specific somatic maintenance	[*ṗM*]	J d^−1^ cm^−3^	29.07	10.27
Volume specific cost of structure	[*E*_*G*_]	J cm^−3^	2800	3156
Fraction of energy used for gametes	*κ*_*R*_	—	0.95	0.99
Shape coefficient	*δ*_*M*_	—	0.23	0.22
Density of structure	*d*_*V*_	g cm^−3^	0.09	0.09
Dry weight-energy coupler	*ρ*_*E*_	g J^−1^	5.71 × 10^−5^	5.71 × 10^−5^
Arrhenius temperature	*T*_*A*_	K	9826	10590
Lower limit of tolerance range	*T*_*L*_	K	273	279.6
Upper limit of tolerance range	*T*_*H*_	K	309	306.1
Arrhenius temperature at lower limit	*T*_*AL*_	K	55400	22670
Arrhenius temperature at upper limit	*T*_*AH*_	K	250600	34540
Metabolic depression constant	*M*_*d*_	—	0.39	0.15

Temperature influences all physiological rates via a thermal sensitivity equation based on Arrhenius thermodynamics^34,35^. During aerial exposure, thermal sensitivity is corrected by a metabolic depression constant, *M*_*d*_ (−) (Table 1).

V is proportional to mussel shell length, *L*_*w*_ (cm), via the shape coefficient (Table 2). Gonadosomatic index, GSI, was computed as gonad dry weight, *W*_gd_ (g), over soma dry weight, *W*_sd_ (g) e.g.^36,37^. The model assumes that gonads are produced continuously during the year of simulations, at which point total aggregate GSI is computed, and after which spawning occurs. Shell-free soma dry weight is composed of the added masses of structure and reserve. Structural mass is derived assuming a specific density, *d*_V_ (g cm^−3^), and reserve mass based on a weight-energy coupler, ρ_E_ (g J^−1^).

### DEB models parameterization

For both species, we estimated parameter values using the program DEBtool_M (available at http://www.bio.vu.nl/thb/deb/deblab/). This program uses the covariation method (Lika 2011), which relies on the Nelder-Mead algorithm to simultaneously fit the various DEB parameters to the species life-history data (see Supplementary Table S1). We included life-history data for subtidal populations only to avoid confounding the effects of aerial exposure. These data were obtained from the literature and our own observations, as follows. Growth data for *Perna* were extracted from Berry (1978) using WebPlotDigitizer 4.0^38^. Growth data for *Mytilus* were determined using 100 mussels collected at Ancona, Italy (N 43°36′56.88″, E 13°31′8.04″), and aged based on growth rings^39^.

### DEB models validation across shore levels

To test the validity of the models under realistic conditions across shore levels, we used the estimated parameter values (Table 3) along with data for environmental drivers (see *Environmental drivers*) to run DEB model simulations for the period between October 2015 and October 2016. Simulations were conducted using an R script^40^, which computes hourly energy flows and dynamics of state variables as a function of body temperature and food availability (see below, Fig. 2, Table 1). We considered tide height as a third environmental driver, defining the alternating periods of aerial exposure and submergence (Fig. 1). During low tide events, the model supresses food ingestion and simulates metabolic depression according to empirical observations conducted in water and air by Tagliarolo and McQuaid^25^ (metabolism is depressed to 0.39 and 0.15 of submerged levels for *Perna* and *Mytilus*, respectively). We did this for three sites separated by 10–30 km on the south coast of South Africa, where both species coexist: Brenton-on-Sea (S 34°04′31.73″, E 23°01′28.06″), Plettenberg Bay (S 34°03′35.81″, E 23°22′50.65″), and Keurboomstrand (S 34°00′17.04″, E 23°27′18.63″). Based on species zonation patterns, we ran simulations for *Mytilus* at high- and mid-shore levels, and for *Perna* at mid- and low-shore levels.

To establish initial values for the model state variables, reserves and structure, we ran spin-up simulations for each species assuming constant temperature (20 °C), food (*f* = 1), and submergence until reaching 4-cm shell length. The initial value for reproductive buffer was set to yield GSI values that matched each species’ lowest value based on our field data (*Perna* = 0.04 and *Mytilus* = 0.12). We ran single simulations for each species at their corresponding shore heights, starting with a 4-cm shell length individual to ensure maturity and to match the size of the biomimetic loggers used to capture body temperatures (see *Environmental drivers*). During the period of this exercise, the reproductive buffer was gradually filled. The maximum value reached was then compared against the observations done on real mussels. We assessed our model predictions based on observations of aggregate annual GSI and maximum shell lengths (see *Gonadosomatic index (GSI) and shell lengths*). We worked with aggregate annual GSI because, while mussel gametogenesis and spawning are often correlated with food and temperature^41^, their exact relationship (or their interaction with other factors) has not been clarified^42,43^. The aim of these simulations was not to trace the observed dynamics in GSI perfectly, but to predict total annual investment in reproduction.

### Environmental drivers

Food availability, body temperature, and exposure/submergence were the DEB model drivers. As a proxy for food availability, we used chlorophyll-a concentration (μg L^−1^) derived from daily Aqua/MODIS (Moderate Resolution Imaging Spectroradiometer) satellite images^16,17^. We used Ocean Color’s web interface (https://oceancolor.gsfc.nasa.gov/cgi/l3) to download daily chlorophyll-a concentrations, estimated based on the OCI algorithm, and provided at a 4-km^2^ spatial resolution. Using SeaDAS 7.1, we extracted 9 pixels daily, centred 15 km offshore from each study site.

Mussel body temperatures were measured using biomimetic temperature loggers i.e. robomussels^44,45^. Loggers (iButton DS1922, Maxim Integrated, CA USA), set to record every 30 min at 0.5 °C resolution, were placed in empty shells (4-cm length) of each species filled with waterproofing electrical resin (3 M Scotchcast 48FR, USA). We deployed two *Perna*-robomussels on the low- and two on the mid-shore, as well as two *Mytilus*-robomussels in the mid- and two on the high-shore levels. Robomussels were cemented to the rock with Z-Spar Splash Compound (Kopper’s Co., USA).

We used robomussel and tide height data to determine when mussels at each shore level were exposed/submerged^46,47^. Hourly tide height predictions were retrieved from XTide (http://www.flaterco.com/xtide/). During rising tide periods, a sudden drop in temperature indicated the time of robomussel submergence, and thus the effective shore level (ESL). This was done for 10 different spring-tide cycles to calculate a mean ESL for each site and shore level. XTide predictions do not account for the influence of wind, atmospheric pressure, or wave splash on the realized tidal inundation time. Thus, to avoid incorrectly regarding exposure as submergence, we added 0.3 m to the ESL (the tidal range on this coast is approximately 2 m). Within the DEB simulation context, this is a conservative approach because it prevents unrealistic spikes in physiological rates due to higher low-tide temperatures that can still be attained while being splashed.

In order to run the DEB simulations, all environmental data were either averaged (body temperature) or linearly interpolated (chlorophyll-a) to an hourly resolution.

### Gonadosomatic index (GSI) and shell lengths

To test the performance of the models across shore levels, we used information on GSI and shell lengths. We collected 30 *Perna* from each of the low- and mid-shore levels, and 30 *Mytilus* from each of the mid-and high-shore levels. We collected animals >30 mm shell length and up to the largest individuals that could be found at each level to cover the full range of adult size. Mussels were preserved in 70% ethanol and taken to the laboratory for subsequent dissection and sizing. We removed epibionts, measured shell lengths with Vernier callipers to the nearest 0.01 mm, and dissected the soft tissue, discriminating between mantle (gonads) and soma. Tissues were dried for 48 h at 60 °C, and weighed to the nearest 0.01 mg. This protocol was repeated 8 times between October 2015 and October 2016, approximately every 1.5–2 months intervals.

Mussel GSI was calculated as gonad tissue dry-weight over soma tissue dry-weight^36^. To compare this with DEB modelled data, we estimated the GSI for an individual of 4-cm shell length based on linear relationships between shell length and GSI computed from the 30 animals collected per species, shore level, site, and sampling date. A standard size of 4-cm was chosen to ensure reproductive maturity and because animals of this size were observed across all sites and shore levels. To compare with DEB-modelled GSIs, empirical GSIs were computed for each species and shore level as annual aggregates by adding the mean peak values observed across the 8 sampling dates. As reported previously^42^, we identified two peaks during the year for both species. Maximum shell lengths at each site and shore level were calculated as the 99^th^ percentiles from all the individuals sampled there.

To assess the model performance we calculated Percent Errors (PE) and Mean Absolute Percent Errors (MAPE) between observations and model predictions. We also regressed observations versus predictions of shell lengths and GSI, and tested whether the slopes were significantly different from expected 1:1 relationships (i.e. slope = 1) using the R package smatr^48^.

## Results

### DEB model parameter values

**Figure 3:**
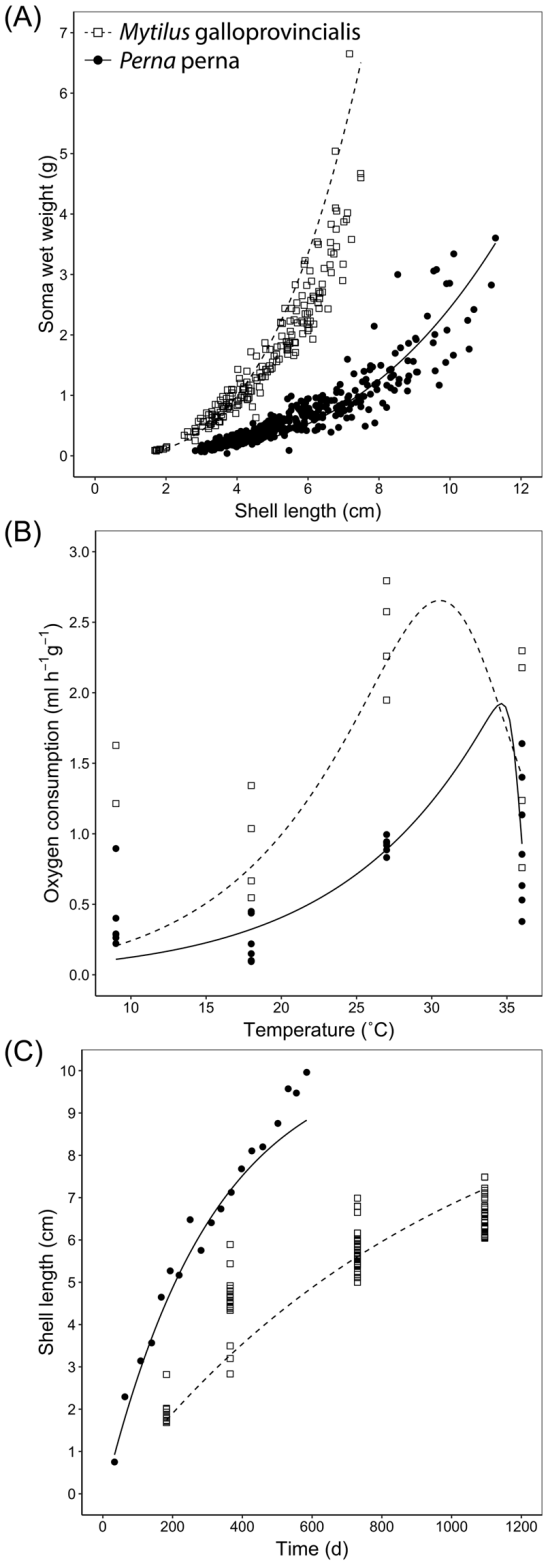
Training data used to parameterize Dynamic Energy Budget models for *Perna perna* and *Mytilus galloprovincialis*. See Table 3 for underlying parameter values. The lines represent model predictions. Supplementary Table S1 provides error estimates for these and other predictions of these species’ life histories.

Using life-history data gathered from the literature and our personal observations (Supplementary Table S1), we successfully parameterized Dynamic Energy Budget models for *Perna* and *Mytilus* (Table 3). These parameters provide good predictions of both species’ allometric scalings, metabolic rates, growth trajectories (Fig. 3), and annual gonadosomatic indices assuming permanent submergence (Supplementary Table S1). The only trait that was not adequately captured by the models was length at larval metamorphosis, because of incomplete data on the larval stages.

The estimated parameter values reflect functional differences between *Perna* and *Mytilus* (Table 3). Most notably, the proportion of catabolized energy allocated to somatic maintenance and growth, *κ*, was substantially lower for *Mytilus*, reflecting its high investment into reproduction.

### Dynamics in environmental conditions

The environmental conditions experienced by *Mytilus* and *Perna* depended on the estimated ESLs, and the corresponding aerial exposure. Generally, these did not vary widely across sites. The ESLs for low-, mid-, and high-shore mussels were 0.91, 1.04, and 1.21 m above mean low lower mark (MLLW) at Brenton-on-sea; 0.85, 1.01, and 1.11 m above MLLW at Plettenberg Bay; and 0.91, 1.01, and 1.15 m above MLLW at Keurboomstrand. The duration of aerial exposure increased from low- to high-shore, with some inter-site variability. The percent times exposed to aerial conditions for low-, mid- and high-shore mussels were 34.41, 43.47, and 53.83% at Brenton-on-sea; 29.71, 41.39, and 47.53% at Plettenberg Bay; and 34.41, 41.39, and 65.40% at Keurboomstrand.

**Figure 4:**
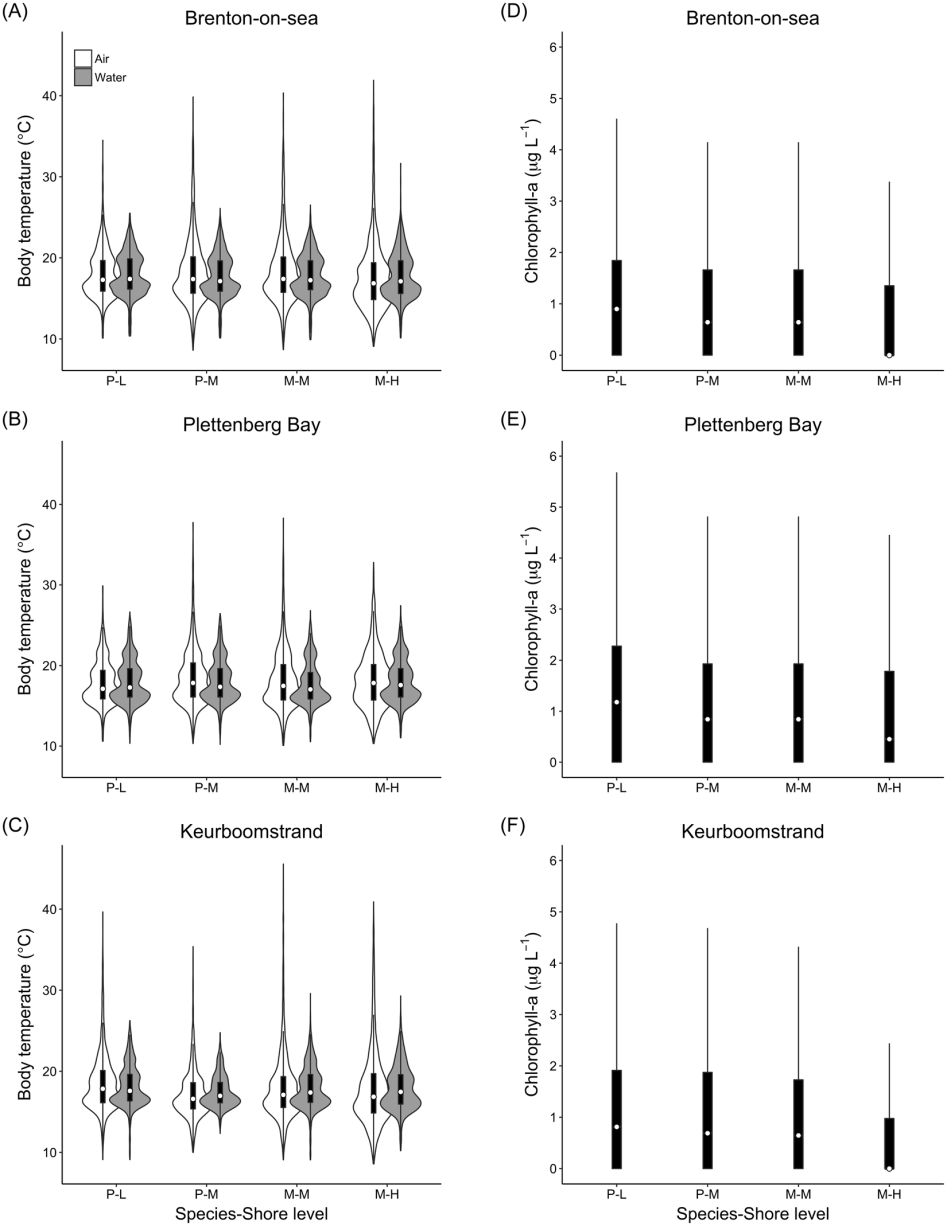
Body temperature (recorded *in situ* using ‘robomussels’, panels A–C) and chlorophyll-a (derived from satellite images, panels D-F) experienced by *Perna perna* and *Mytilus galloprovincialis* during the study period (October 30^th^ 2015 to October 30^th^ 2016) at each site (Brenton-on-sea, Plettenberg Bay, Keurboomstrand). Data are provided as a function of species and shore level: *Perna*-Low (P-L), *Perna*-Mid (P-M), *Mytilus*-Mid (M-M), and *Mytilus*-High (M-H). Body temperatures regarded as aerial or submerged in the DEB model simulations are plotted separately. Chlorophyll-a plots are aggregates of aerial and submerged periods, with values of zero assigned to the former. The white circles are the medians. The boxes mark the 25^th^ and 75^th^ percentile of the distributions. The vertical lines are 1.5 time the interquartile ranges. Violin shapes show the distribution densities of the body temperature data. Violins are not shown for chlorophyll-a data because outliers would squeeze the boxplots rendering them unintelligible.

Median body temperatures were very similar across sites, species, shore levels, and medium (air/water). The temperature range, however, differed between media, and among shore levels (Fig. 4A–C). The range was consistently higher during periods of aerial exposure, especially because of a higher frequency of high temperatures. The interquartile range of aerial body temperatures increased towards the high-shore at every site (Fig. 4A–C). At Plettenberg Bay and Keurboomstrand, maximum aerial temperatures were higher on the mid- than high-shore, which is likely explained by the mediating effect of neighbouring boulders on direct solar radiation. Some body temperatures categorized as submerged based on ESLs were apparently too high, notably high-shore *Mytilus* at Brenton-on-sea where some records exceeded 30 °C (Fig. 4A). Most of our submerged body temperatures, however, fell substantially lower than the historical daily maximum seawater temperatures recorded at Knysna (~4 km from Brenton-on-sea) and Plettenberg Bay (24.7 and 24.6 °C, respectively) by the South African Coastal Temperature Network, SACTN.

Satellite-derived chlorophyll-a estimates (a proxy for food) were also similar between sites, with overall median values around 1 μg L^−1^ (Fig. 4D–F). Strong variability over the year was observed for all sites, especially Plettenberg Bay. The temporal dynamics in chlorophyll-a appeared coupled between the neighbour sites Plettenberg Bay and Keurboomstrand, but less so with Brenton-on-sea (Supplementary Fig. S6). Due to increasing aerial exposure with shore level, high-shore mussels received less food in the DEB models than low-shore mussels. This gradient was more evident at Brenton-on-sea and Keurboomstrand than Plettenberg Bay (Fig. 4D–F).

### Gonadosomatic index (GSI) and shell lengths

**Figure 5:**
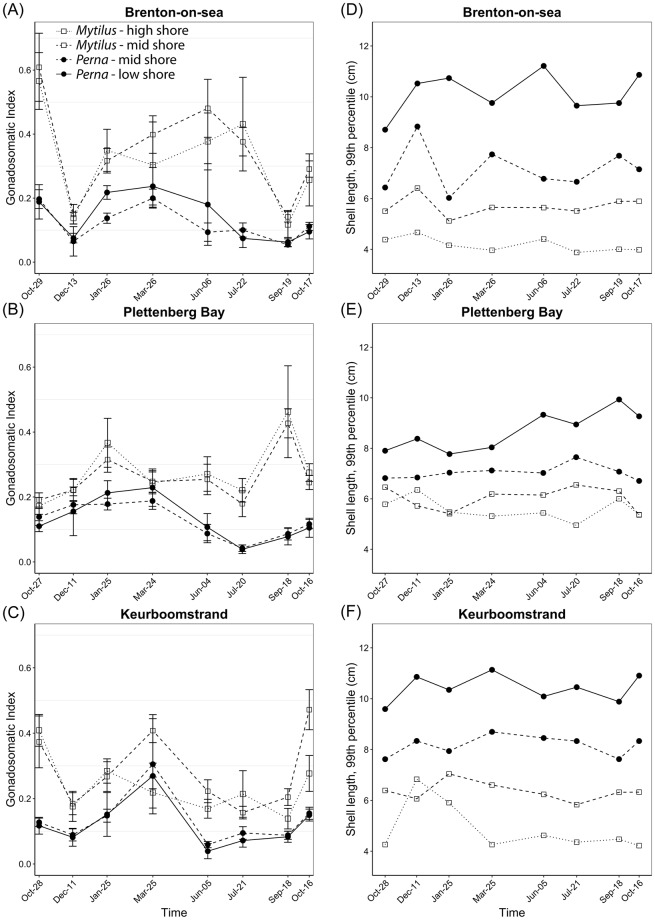
Observed gonadosomatic index (GSI, panels A–C) and maximum shell length (panels D–F) across time for *Perna perna* and *Mytilus galloprovincialis* at each site (Brenton-on-sea, Plettenberg Bay, Keurboomstrand) and shore level. GSI data were estimated means ± 95% CI for a 4-cm shell length mussel, computed from linear models relating shell length and gonad dry weight. Maximum shell lengths were calculated as the 99^th^ percentile of all mussels measured across the study period. Symbols: dots = *Perna perna*; squares = *Mytilus galloprovincialis*. Connecting lines: continuous = low-shore; dashed = mid-shore; dotted = high-shore.

GSI cycled over the year at every site (Fig. 5A–C). It was always higher for *Mytilus* than *Perna*. For each species, GSI was similar across shore levels, but when differences were observed, the GSI values were higher on the lower shore. For *Mytilus*, two peaks in GSI could be detected, generally around March and October. *Perna* showed a higher peak in March, and a lower one in October (Fig. 5A–C).

Observed maximum mussel size also varied with site, species, and shore level (Fig. 5D–F). Mussels were on average smaller at Plettenberg Bay than Brenton-on-sea and Keurboomstrand. *Perna* was consistently larger than *Mytilus*, even when compared at the same mid-shore level. Individuals were larger towards the low-shore (Fig. 5D–F).

### DEB model validation

**Figure 6:**
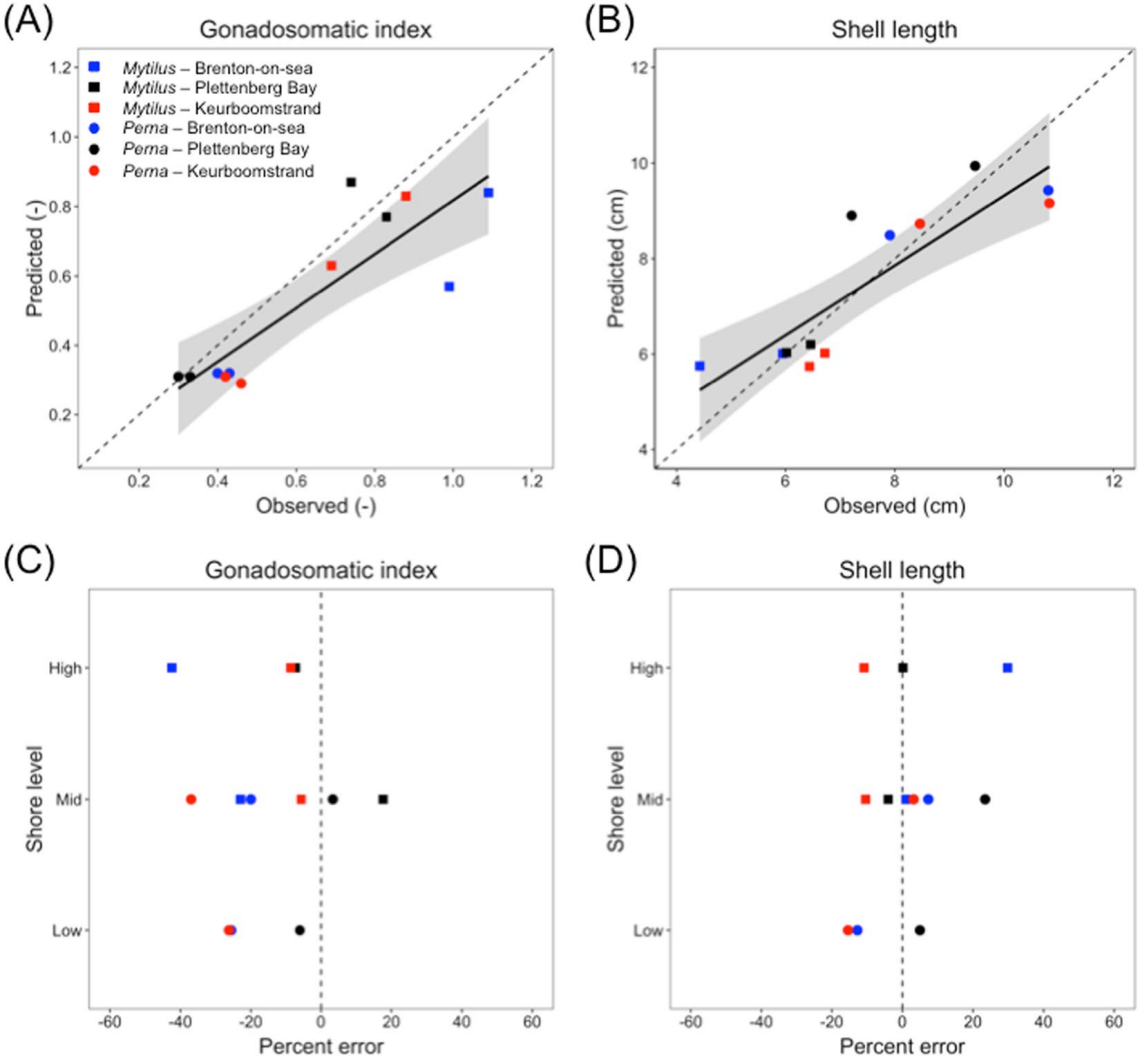
Comparison of Dynamic Energy Budget (DEB) model predictions with field observations of (**A**) mean gonadosomatic index (GSI) and (**B**) shell length. Panels (C,D) provide the percent errors calculated based on these predictions and observations across shore levels for each species and site. Dashed lines indicate a perfect match. Linear regression fits (±95% CIs) are provided in panels (A,B).

We found significant correlations between expected values and observations for both annual GSI and maximum shell length (Pearson’s correlation: GSI, ρ = 0.87, p < 0.001; shell length, ρ = 0.87, p < 0.001). Additionally, observations and predictions for both traits were not significantly different from expected 1:1 relationships (slope test: p > 0.05) (Fig. 6A,B).

On average the models predicted shell lengths better than GSIs (MAPE = 10.3 and 18.6%, respectively) (Fig. 6). The few unsatisfactory predictions of shell length were observed at the mid- and high-shore levels (Fig. 6D). We observed poor predictions of GSI except at Plettenberg Bay. At Brenton-on-sea and Keurboomstrand, GSI was consistently underestimated across shore levels for both species (Fig. 6C). No obvious patterns of model error were detected between species (Fig. 6; see Supplementary Table S2 for details of model errors by site, shore level, species, and trait).

**Figure 7:**
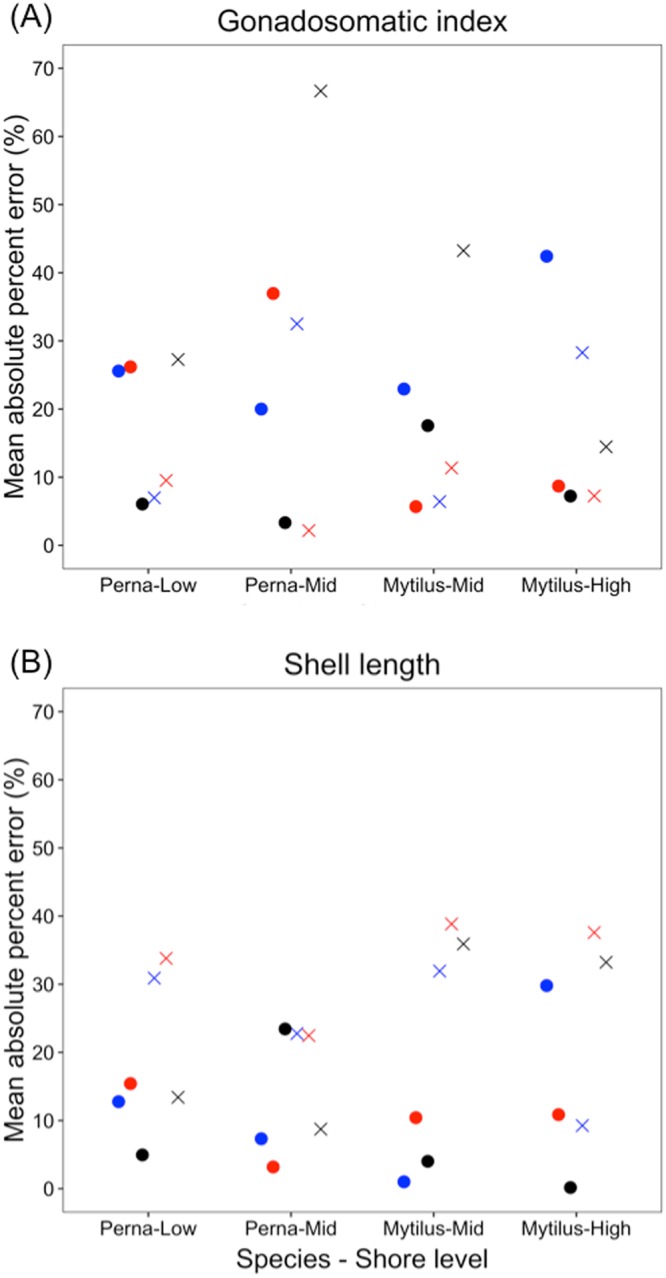
Mean absolute percent errors (MAPE) of model predictions of (**A**) gonasomatic index and (**B**) shell length performed either considering metabolic depression during aerial exposure (points) or not (crosses). Colours represent sites: blue = Brenton-on-sea; black = Plettenberg Bay; red = Keurboomstrand.

To test the value of implementing metabolic depression in the model directly, we compared the model skill between simulations run with and without the metabolic depression parameter. Metabolic depression improved predictions of both maximum shell length and GSI, as not considering it would yield average MAPEs of 26.6 and 21.3% respectively. Improvement in predictions varied with trait, species, and shore level (Fig. 7). For example, at Brenton-on-sea maximum, shell length was generally better predicted than GSI when including metabolic depression. However, for mid-shore *Perna*, while metabolic depression improved predictions of shell length it worsened those of GSI.

## Discussion

One of the main reasons for the growing popularity of Dynamic Energy Budget theory in the ecological literature is its ability to model organismal responses to a variable environment on a mechanistic basis. Its utility has been extensively proven in comparisons of systems where conditions differ across latitudes, or across eco-regions^49,50^. However, implementing DEB models for populations that experience regular, strong fluctuations in abiotic factors has received considerably less attention. Here we test the efficacy of DEB models for intertidal populations of the mussels *Perna perna* and *Mytilus galloprovincialis*. These species are important ecological engineers that occupy a large portion of rocky shores throughout temperate and subtropical regions.

We followed a two-step approach to finding appropriate parameters to model the energy budget of intertidal populations. First, using the covariation method in DEBtool^51^, we estimated the primary parameters that would define the species’ physiological responses under standard, aquatic conditions. These parameters provided adequate fits for allometric scalings, metabolic rates, growth trajectories, and reproduction for both *Mytilus* and *Perna*. Furthermore, the parameter values are consistent with known differences in life-history traits between these species. For instance, given differences in the allocation parameter κ, the models capture the higher investment of *Mytilus* into reproduction, one of the key traits that have contributed to the ability of this species to become invasive on all continents except Antarctica^52^. Additionally, both earlier empirical studies^53^ and the data used to parameterize the models showed that, under equal environmental conditions, standard metabolic rate and GSI are higher for *Mytilus* than *Perna*. Note that, while *Mytilus* is also expected to grow faster than *Perna*^53,54^, we were unable to find growth data collected under the same standard conditions (e.g. seawater temperatures experienced by *Perna* in the published studies were warmer, accelerating its growth rate). However, this is not a problem for the parameterizing exercise if information on temperature and food is accounted for while running DEBtool^51^.

Second, to examine the possibility of applying these models under extremely variable conditions, we validated them using independent datasets on maximum mussel shell length (derived from structural length) and accumulated annual GSI, along with detailed information on the dynamics of food availability and body temperatures experienced by animals across the shore. Earlier work by Sarà, *et al*.^11^ provided initial confirmation that DEB models could be used to simulate dynamics in physiological performance of Mediterranean Sea intertidal *Mytilus* populations, and predict the sites where mussels could grow and survive. That study also accounted for the suspension of feeding during emersion, but ignored metabolic depression. Here we took one step further and introduced a simple constant to consider at a gross level the various underlying physiological processes that take place during aerial exposure^27–30^. Although still imperfect (see *Dynamics in environmental conditions and model performance*), this approach improved predictions of mussel growth across the shore, and we therefore recommend explicitly incorporating metabolic depression where appropriate, including situations involving aestivation or hibernation as well as intertidal DEB models. For intertidal systems, including metabolic depression is especially critical where species distributions extend higher on the shore due to strong negative biotic interactions on the low-shore e.g.^54–57^.

Accurate estimates of life-history traits are contingent upon the level of detail with which environmental drivers are characterized^58^. In general, our model outputs agreed with expectations (i.e. physiological condition was better for lower-shore mussels), indicating that environmental gradients due to tidal variability were adequately captured. However, the fact that the model validations consistently underestimated annual GSI points to the possibility of unaccounted sources of error. We now discuss possible sources of error, specifically potential inaccuracies associated with the data on tidal height dynamics, body temperature and food, and over-estimates of observed reproductive output. We suggest that the latter is likely the main cause for the models’ suboptimal performance here.

The performance of these models is tightly dependent on our ability to track periods of aerial exposure, as these directly affect the amount and rates of energy ingested/assimilated by mussels, as well as the rate of energy allocation to maintenance, growth, and reproduction^8,31^. Here we determined periods of aerial exposure by combining tidal height predictions and *in situ* temperature data, a method that has been advocated in the literature, as it simplifies data collection and allows the sampling of more sites^46,47^. Our observation that temperature was more variable in air than water suggests that on average, we identified periods of submergence correctly. However, because we used tidal height predictions and not observations, the influence of wind or atmospheric pressure on wave height and the realized exposure time of individuals could not be estimated. The fact that supposedly submerged body temperatures occasionally reached abnormally high temperatures suggests occasional misidentification of the beginning or end of periods of aerial exposure. Indeed, the example data illustrated in Fig. 1 showed that for three consecutive days, while body temperature rose with the ebb of the tide, it did not decrease at the presumed time of re-submergence. Future applications of the model could consider alternative methods to record exposure directly; for example, by coupling temperature and pressure loggers, Mislan, *et al*.^59^ were able to trace exposure, submergence, and surge periods with great accuracy.

By using species-specific robomussels in this study, we captured body temperature dynamics more accurately than via proxies like air temperature^44^. Although we did not observe obvious differences in body temperature between mid-shore *Mytilus* and *Perna*, previous studies suggest that shell colour, material properties or the presence of epibionts can drive variation within and among species, with potentially important physiological and ecological consequences^60–62^.

Chlorophyll-a has long been used as a proxy of food availability for filter feeders, including within a DEB framework^33^. Here we worked with Aqua/MODIS satellite-derived estimates because data are readily available and offer an opportunity for models to be employed globally. The caveat is that, while satellite-derived estimates have been validated as indicators of coastal phytoplankton and organic material in other regions^11,63^, no such relationship exists for South African shores. Our approach may ignore important sources of variability in the quantity and quality of food available to mussels, including the effects of tidal or topographical hydrodynamics, local upwelling, or sediment loads^64–66^. Either conducting the necessary validations for satellite data, or better, measuring food availability *in situ* would certainly improve the application of DEB models across the shore.

The environmental variables tested contributed to the overall model skill, and our validation revealed that reproductive output was the only trait that was poorly predicted. Given that all shore levels and both species were affected by this problem, it is unlikely that it can be resolved by improving environmental data or by tweaking the model parameters. A more plausible explanation is that our empirical observations may have been misleading. In the study region, mussel GSI can cycle irregularly over the year, and often spawning is not complete^42^. Because the ecophysiological mechanisms behind this reproductive strategy are not fully understood^42^, we did not incorporate them in this model. For simplicity, we assumed that mussels would release all their gametes once a year, which could have led to overestimates of the calculated annual GSI. This is an area that deserves further attention, especially if we are to use these models to predict population level dynamics.

With ongoing technological developments in sensor and logging devices, we can track environmental conditions with increasing precision and across a broad range of spatial/temporal scales^2,67^. As a result, mechanistic models potentially form excellent tools for predicting the responses of organisms to different environmental conditions. Because conditions are normally in a state of constant flux, however, these models need to incorporate natural environmental variability. Our results show that DEB models are capable of incorporating such variability and making good predictions for growth across a particularly steep ecotone. The underestimation of reproductive output by our models highlights the importance of fine features of biology, which can readily be incorporated, and potentially the importance of variability in the abiotic conditions experienced at the individual level^2^. For example, experimental modification of *in situ* flow dynamics at centimetre scales has been shown to affect mussel growth rates^64^.

Intertidal habitats are amongst the most productive and diverse ecosystems on Earth, but they are also among the most environmentally variable. The demonstrated ability of our models to incorporate this variability into accurate predictions of species traits illustrates the effectiveness of the mechanistic approach of DEB to predicting responses under dynamic conditions. Such models can also help predict the responses of organisms to expected climate change scenarios, under which both air and sea surface temperatures are expected to vary^4,68^.

## Electronic supplementary material

Supplementary material

## Data Availability

All data are available in the manuscript and supplementary material.
