# Supplementary material for: Applicability of Dynamic Energy Budget (DEB) models across steep environmental gradients
Source: Sci Rep. 2018 Nov 6;8:16384. doi: 10.1038/s41598-018-34786-w (PMC6219521; doi:10.1038/s41598-018-34786-w)
Supplement: Supplementary file 1 — Supplementary material [file 41598_2018_34786_MOESM1_ESM.pdf]

Applicability of Dynamic Energy Budget (DEB) models across steep environmental gradients

Cristián J. Monaco* & Christopher D. McQuaid
Department of Zoology and Entomology, Rhodes University, Grahamstown, South Africa
* Present address: Southern Seas Ecology Laboratories, School of Biological Sciences, The University of Adelaide, Adelaide, SA 5005, Australia
Corresponding author: Cristián J. Monaco, cristianmonaco@gmail.com

Supplementary material

Supplementary Table S1 Life-history data used to parameterize Dynamic Energy Budget (DEB) models for *Perna perna* and *Mytilus galloprovincialis* in the program DEBtool_M, and corresponding predictions. Model fits are examined based on Absolute Percentage Errors (APE).

Life-history data	*Perna*		*Mytilus*	
	Obs (Pred)	APE %	Obs (Pred)	APE %
Age at birth, d	2[1] (2.22)	11.00	1[2] (1.15)	15.00
Age at metamorphosis, d	12[1] (10.02)	16.50	27[2] (27.01)	0.04
Age at puberty, d	100[3] (96.18)	3.82	210[4] (223.9)	6.62
Length at birth, μm	88.22[1] (88.64)	0.48	100[2] (99)	1.00
Length at metamorphosis, μm	255[1] (1071)	320.00	272[2] (896.7)	229.67
Length at puberty, cm	2.90[5] (2.98)	2.76	3[4] (3.14)	4.67
Ultimate length, cm	12[6] (12.46)	3.83	15[7] (16.09)	7.27
Gonadosomatic index	0.60[6] (0.61)	1.67	2[8] (1.58)	21.00
Length vs. soma wet weight	Fig. 1a*	28.24	Fig. 1d*	18.97
Time vs. length	Fig. 1b[3]	6.34	Fig. 1e*	13.22
Temperature vs. O_2 consumption	Fig. 1c[9]	35.15	Fig. 1f[9]	32.37
	$\bar{x}$	39.07	$\bar{x}$	31.80

Superscript numbers next to observed data are sources listed in the Reference section.

* Indicates that the authors collected the data.

Supplementary Table S2 Dynamic Energy Budget (DEB) model predictions mean absolute percent errors (MAPE) based on field observations of (A) mean gonadosomatic index (GSI) and (B) maximum shell length. Calculations are given for *Perna* and *Mytilus* inhabiting different shore levels at Brenton-on-Sea, Plettenberg Bay, and Keurboomstrand. MAPE averaged across shore levels and sites are also provided. Observed GSI is the sum of the two peaks observed during the year of measurements (Fig. 4). Simulations to predict maxima lengths were carried until reaching the saturation part of the growth curve by repeating the environmental drivers data over an assumed life span of 10 years.

A) Gonadosomatic Index					
	Perna perna		*Mytilus galloprovincialis*		
	Low	Mid	Mid	High	x̄
Brenton	32.56	30.00	32.11	52.53	36.80
Plettenberg bay	12.12	6.67	4.05	19.28	10.53
Keurboomstrand	30.95	43.48	17.05	24.64	29.03
x̄	25.21	26.71	17.74	32.15	
B) Shell length					
	Perna perna		*Mytilus galloprovincialis*		
	Low	Mid	Mid	High	x̄
Brenton	12.77	7.33	1.01	29.80	12.73
Plettenberg bay	4.96	23.44	4.02	0.17	8.15
Keurbooms	15.42	3.19	10.42	10.87	9.87
x̄	11.05	11.32	5.15	13.61	

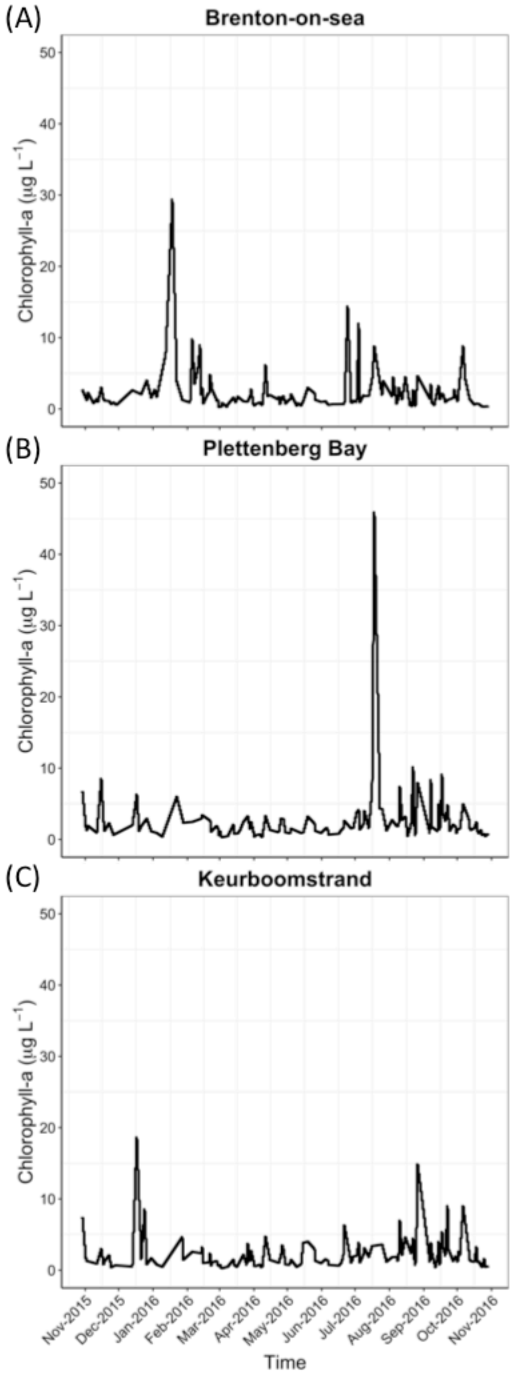

Supplementary Fig. S1. Chlorophyll-a time-series data (derived from satellite images) used as driving variables in the DEB models of *Perna perna* and *Mytilus galloprovincialis* during the study period (October 30th 2015 to October 30th 2016). Data from Brenton-on-sea, Plettenberg Bay, and Keurboomstrand are given in panels A-C.

References

1. L. Aarab *et al.*, Embryonic development and influence of egg density on early veliger larvae and effects of dietary microalgae on growth of brown mussel *Perna perna* (L. 1758) larvae under laboratory conditions. *Aquac Int* **21**, 1065 (2013).
2. C. Sánchez-Lazo, I. Martínez-Pita, Effect of temperature on survival, growth and development of *Mytilus galloprovincialis* larvae. *Aquac Res* **43**, 1127 (2012).
3. P. F. Berry, "Reproduction, growth and production in the mussel, *Perna perna* (Linnaeus), on the east coast of South Africa" *0869890239* (Oceanographic Research Institute, 1978).
4. V. U. Ceccherelli, R. Rossi, Settlement, growth and production of the mussel *Mytilus galloprovincialis*. *Mar Ecol Prog Ser* **16**, 173 (1984).
5. T. E. Phillips, Rhodes University (1995).
6. V. Acosta *et al.*, Differential growth of the mussels *Perna perna* and *Perna viridis* (Bivalvia: Mytilidae) in suspended culture in the Golfo de Cariaco, Venezuela. *J World Aquacult Soc* **40**, 226 (2009).
7. R. M. Harbo, *Shells and shellfish of the Pacific Northwest, a field guide*. (1997), pp. 278.
8. C. Van Erkom Schurink, C. L. Griffiths, A comparison of reproductive cycles and reproductive output in four southern African mussel species. *Mar Ecol Prog Ser* **76**, 123 (1991).
9. M. Tagliarolo, C. McQuaid, Sub-lethal and sub-specific temperature effects are better predictors of mussel distribution than thermal tolerance. *Mar Ecol Prog Ser* **535**, 145 (2015).
